# Enhanced Visible Light-Driven Photocatalytic Water-Splitting Reaction of Titanate Nanotubes Sensitised with Ru(II) Bipyridyl Complex

**DOI:** 10.3390/nano13222959

**Published:** 2023-11-16

**Authors:** Mauro Malizia, Stuart A. Scott, Laura Torrente-Murciano, Adam M. Boies, Talal A. Aljohani, Herme G. Baldovi

**Affiliations:** 1 Department of Chemical Engineering and Biotechnology, University of Cambridge, Philippa Fawcett Drive, Cambridge CB3 0AS, UK; mauro.malizia.86@gmail.com (M.M.); lt416@cam.ac.uk (L.T.-M.); 2 Department of Engineering, University of Cambridge, Trumpington Street, Cambridge CB2 1PZ, UK; sas37@eng.cam.ac.uk (S.A.S.); amb233@eng.cam.ac.uk (A.M.B.); 3 Refining and Petrochemical Technology Institute, King Abdulaziz City for Science and Technology, Riyadh 11442, Saudi Arabia; taljohani@kacst.edu.sa

**Keywords:** titanate nanotubes, ion exchange, tris(2,2′-bipyridine)ruthenium(II), photocatalysis, water splitting, solar simulated light, hydrogen evolution reaction, dye-sensitized photocatalyst

## Abstract

The ion exchange of Na^+^ cations was used to photosensitise titanates nanotubes (Ti-NTs) with tris(2,2’-bipyridine)ruthenium(II) cations (Ru(bpy)_3_^2+^); this yielded a light-sensitised Ti-NTs composite denoted as (Ru(bpy)_3_)Ti-NTs, exhibiting the characteristic absorption of Ru(bpy)_3_^2+^ in visible light. Incident photon-to-current efficiency (IPCE) measurements and the photocatalytic reduction of methyl viologen reaction confirmed that in the photosensitisation of the (Ru(bpy)_3_)Ti-NTs composite, charge transfer and charge separation occur upon excitation by ultraviolet and visible light irradiation. The photocatalytic potential of titanate nanotubes was tested in the water-splitting reaction and the H_2_ evolution reaction using a sacrificial agent and showed photocatalytic activity under various light sources, including xenon–mercury lamp, simulated sunlight, and visible light. Notably, in the conditions of the H_2_ evolution reaction when (Ru(bpy)_3_)Ti-NTs were submitted to simulated sunlight, they exceeded the photocatalytic activity of pristine Ti-NTs and TiO_2_ by a factor of 3 and 3.5 times, respectively. Also, (Ru(bpy)_3_)Ti-NTs achieved the photocatalytic water-splitting reaction under simulated sunlight and visible light, producing, after 4 h, 199 and 282 μmol×H_2×_g_cat_^−1^. These results confirm the effective electron transfer of Ru(bpy)_3_ to titanate nanotubes. The stability of the photocatalyst was evaluated by a reuse test of four cycles of 24 h reactions without considerable loss of catalytic activity and crystallinity.

## 1. Introduction

Titanium dioxide, among other metal oxide semiconductors such as Fe_2_O_3_ or BiVO_4,_ is one of the most widely used photocatalysts for solar water-splitting [1,2]. Ever since Fujishima and Honda [3] reported the ability of TiO_2_ to split water upon irradiation with UV light, there has been considerable interest in expanding the high intrinsic photocatalytic activity of TiO_2_ and related materials into the visible region [4,5]. Various approaches have been developed to expand the photoresponse of TiO_2_, including an understanding of the influence of particle size, surface area, particle morphology, and crystal structures on the band gap of the material [5,6,7]. Alternatively, extending the photoactivity of titanium oxide and related materials towards the visible region has been addressed by sensitising with plasmonic nanoparticles [8,9,10] and introducing extra electronic levels by doping the titanium dioxide crystal [11,12] or by forming metal oxide heterojunctions [1,13]. titanate nanotubes (Ti-NTs) are widely used to split water because of their ordered tubular structure, ion-exchange ability, and, compared to TiO_2_, the longer lifetime of the photogenerated electron–hole pairs [14,15]. However, the photocatalytic efficiency of Ti-NTs in absolute terms is still below the theoretical maximum owing to the fast electron–hole pair recombination and the lack of photoresponse with visible irradiation [16]. The research has been especially directed at extending the photoactivity of Ti-NTs to the visible region [9,17,18]. The ion-exchange ability of Ti-NTs offers a method of anchoring metallic cations [19], molecules, and metallic complexes on their external surface to alter the optical, magnetic, and electric properties of pristine Ti-NTs. The resulting modified Ti-NTs have been proposed for several applications, e.g., molecular or biological sensors [20], LEDs [21], dye-sensitised solar cells [22,23,24], catalysis [25,26,27], and photocatalysts [6,7,17].

Dye-sensitised solar cells are produced when a semiconductor is sensitised with organic molecules to enhance optical absorption towards the visible-light region or to improve the efficiency of the electronic mobility, improving the performance of the cell. The most popular dye-sensitised solar cells use TiO_2_ nanoparticles with attached metal complexes, such as tris(2,2’−bipyridine) ruthenium(II), (Ru(bpy)_3_^2+^) [21,24,28]. Accordingly, researchers have used ruthenium polypyridyl complexes to promote the photocatalytic activity of TiO_2_ nanoparticles for hydrogen generation in the presence of sacrificial electron donors. Gratzel et al. [29] used suspensions of Ru polypyridyl complexes adsorbed on TiO_2_ in the presence of colloidal Pt nanoparticles, acting as a hydrogen evolution reaction catalyst, to enhance the water-splitting reaction. In more elaborated systems, Mallouk and co-workers [30] used ruthenium polypyridyl–viologen, which was adsorbed at the entrances to the pores in mordenite containing TiO_2_ clusters and Pt nanoparticles. These photoelectrodes were applied to photoelectrocatalytic water-splitting in the presence of tertiary amines, producing hydrogen under visible-light irradiation. Garcia et al. [31] also reported the use of ruthenium trispyridyl and other metal complexes to photosensitise graphene oxide semiconductors with visible light for photocatalytic applications. While these precedents establish the ability of ruthenium trisbipyridyl complexes to photosensitise TiO_2_ and other semiconductors, a similar strategy has not been applied to other Ti-based semiconductors, such as titanates. The purpose of the present paper is to address this situation by exploring the use of Ru(bpy)_3_^2+^ to photosensitise titanate nanotubes for use in the generation of hydrogen from water.

Absorption bands of ruthenium polypyridyl complexes are dominated by metal-to-ligand charge transfer (MLCT) electronic transitions that could serve to promote the injection of electrons into the conduction band of semiconductors., see Scheme S1. For instance, in the visible region, light absorption at 460 nm allows the ^1^(dπ^6^) → ^1^(dπ^5^π*^1^) transition. Another absorption transition of Ru(bpy)_3_^2+^ responsible for MLCT in the visible region appears at 515 nm, which arises from the electronic transition from the ground state (^1^(dπ^6^)) to the triplet state (^3^(dπ^5^π*_2_^1^)). This complex has its more energetic MLCT electronic transition located in the near-UV (300–400 nm) region, in which the ground state(^1^(dπ^6^)) passes to the ^3^(dπ^5^π*_2_^1^) excited state [32,33]. These states have both oxidising (dπ_Ru_^5^) and reducing sites (π_bpy_*^1^) on the same molecule and provide a formal redox potential of −0.92 V and 0.96 V for the pairs [Ru(bpy)_3_^3+^/Ru(bpy)_3_^2+^*] and [Ru(bpy)_3_^2+^*/Ru(bpy)_3_^+^], respectively, in 0.1 M KNO_3_ vs. NHE [32,33].

The rate of hydrogen production in Ti-NT materials varies depending on whether light-harvesting complexes are attached, whether or not a co-catalyst is present, and the light source and composition and concentration of the sacrificial donor [29,34]. When exposed to the full spectrum of a xenon or mercury lamp, the maximum reported rates of hydrogen production range from 1.5 to 3.0 mmol×g^−1^×h^−1^ when using Ti-NTs to which a noble metal such as platinum has been attached [35,36,37]. However, these values markedly drop to values between 11.7 and 29.2 μmol×g^−1^×h^−1^ when the same material is irradiated only by visible light (>430 nm cut-off light filter) [38,39,40].

This paper demonstrates that cationic ruthenium dyes can photosensitise Ti-NTs for photocatalytic hydrogen evolution and water-splitting reactions using an inexpensive method of preparation. Specifically, anionic trititanate was prepared, possessing a large specific surface area [26,41], a tubular morphology, and different Na^+^ content to be exchanged by the dye Ru(bpy)_3_^2+^. The resulting composite has not previously been reported as a photocatalyst for hydrogen evolution and water splitting; therefore, considering the importance of TiO_2_ as a semiconductor, it is worthwhile to investigate its activity for solar water splitting.

## 2. Experimental

### 2.1. Samples Preparation

*Synthesis of NaHTi_3_O_7_, (NaH)Ti-NTs* [41,42,43,44]. In a typical Ti-NTs preparation, 5 g of a commercial TiO_2_ anatase (Sigma Aldrich) with particle size between 80 and 200 nm was suspended in 80 mL of aqueous 10 M NaOH. The suspension was vigorously stirred for 30 min before submitting the sample to hydrothermal treatment at 150 °C in a sealed Teflon autoclave for 30 h. After this time, the supernatant was removed, and the solid was suspended in 80 mL 0.1 M H_2_SO_4_ and stirred gently for 30 min at room temperature. The solid was collected by vacuum filtration using a 0.22 μm nylon filter and dispersed again in 80 mL of deionised water at 80 °C, stirring slowly for 2 h. Finally, the nanotubes were filtered in vacuo and dried at 80 °C in an oven under reduced pressure for 12 h. The resultant solid was spread on a pH paper, and a drop of water was added to the solid, revealing a pH of 7. As a result, NaHTi_3_O_7_ was obtained as the desired product with a 76% yield.

*Synthesis of H_2_Ti_3_O_7_ and Na_2_Ti_3_O_7_, ((H)Ti-NTs and (Na)Ti-NTs)* [45]. A freshly prepared Ti-NTs (0.5 g) were suspended in an acidic aqueous solution (2 M H_2_SO_4_) to prepare H_2_Ti_3_O_7_ or in a basic aqueous solution (2 M NaOH) to prepare Na_2_Ti_3_O_7_, and stirred gently for 2 h at 80 °C. The solid was washed with fresh deionised water and recovered by filtration under vacuum using a 0.22 μm nylon filter. The solid was washed with deionised water until the washings became neutral pH. Finally, the nanotubes were dried at 80 °C in a vacuum oven. The dried solid was spread onto pH indicator paper, and with a drop of water, the pH paper changed in colour, showing acid or basic for H_2_Ti_3_O_7_ or Na_2_Ti_3_O_7_, respectively. In this synthetic step, yield varied from H_2_Ti_3_O_7_ to Na_2_Ti_3_O_7,_ being ~50 and 90%, respectively.

*Synthesis of [Ru(bpy)_3_]_x_Na_2-x_Ti_3_O_7_, (Ru(bpy)_3_)Ti-NTs.* A quantity of 300 mg of Ti-NTs was sonicated for 15 min in 100 mL of deionised water with continuous stirring. Meanwhile, in another vessel, 50 mg of tris(2,2’−bipyridine)ruthenium(II) dichloride was dissolved in 30 mL of distilled water and heated to 80 °C. On reaching this temperature, the tris(2,2’−bipyridine)ruthenium(II) dichloride suspension was added dropwise to Ti-NTs, and the whole slurry was left for stirring to 3 h. After this time, the suspension was cooled to room temperature, and the solid was recovered by vacuum filtration with a 0.22 μm nylon filter. The solid was washed by resuspending it in deionised water and recovered by filtration. This step was repeated until the water became colourless. The nanotubes were dried at 100 °C for 12 h in vacuo. The resulting yellow–orange solid was washed with 24 h Soxhlet of a solution of acetonitrile. Finally, the solid was dried at 100 °C in a vacuum for 24 h with a 60% yield.

### 2.2. Sample Characterisation

*Transmission electron microscopy (TEM)*. To prepare a specimen for TEM studies, a dilute suspension was dispersed in deionised water and sonicated. A drop was added to a holey carbon film on a 400 mesh copper grid (Agar Scientific, Essex, UK) and allowed to dry in the air. High-resolution TEM (HRTEM) images were obtained using a Tecnai F20 system at 200 kV (JOEL Ltd, Tokyo, Japan) with a high-brightness field emission gun (FEG).

*X-ray diffraction analysis*. Analyses of powdered samples were performed with a Bruker D8 Advance (Bruker, Billerica, MA, USA) powder X-ray diffractometer using Cu Kα (λ = 1.54 Å) radiation, operating in reflection, theta-theta mode with a 2D strip detector. The samples were measured utilising an aluminium holder.

*Diffuse reflectance UV-Vis spectroscopy*. The samples were measured as a dry powder, and their diffuse reflectance spectra were carried out on an Agilent Cary 300 spectrophotometer (Agilent Technologies, Santa Clara, CA, USA) coupled to a 70 mm Cary Diffuse Reflectance Accy Internal integrating sphere and using a BaSO_4_ block as a standard.

*Fluorescence spectroscopy*. Fluorescence spectra were measured using 10 mm × 10 mm quartz cuvettes of 4 mL total volume in a Jacso FP-8500 spectrofluorometer (Jasco International Co., Ltd., Tokyo, Japan). First, suspensions of 1.2 mg of (Ru(bpy)_3_)Ti-NTs and (Na)Ti-Nts in 3 mL of acetonitrile of HPLC grade were prepared and purged with argon for 15 min. The optical absorption of this sample was measured to be 0.42 a.u. at 460 nm. Then, a suspension of Na_2_Ti_3_O_7_ was prepared with the same optical absorption. According to elemental analysis, the estimated Ru(bpy)_3_Cl_2_ concentration in (Ru(bpy)_3_)Ti-NTs dispersion was 0.0102 mg in 3 mL of acetonitrile, equivalent to 1.36 × 10^−6^ M. Argon was bubbled through the dispersions for 10 min before the measurement in order to remove air. For relative quantum emission efficiency calculations, a 3 mL standard solution of 1.36 × 10^−6^ M of Ru(bpy)_3_Cl_2_ in acetonitrile was prepared and purged with argon for 15 min prior to measurements being made.

*Elemental analysis.* The content of sodium and ruthenium complex in Ti-NTs samples were measured by inductively-coupled plasma optical emission spectroscopy (ICP-OES) using a Perkin Elmer Optima 2100 DV instrument (PerkinElmer, Inc. MA, USA). The samples were digested in aqua regia, and the resulting solutions were diluted with ultrapure water and analysed by the ICP-OES. The instrument was calibrated beforehand using a suitable ICP standard solution.

### 2.3. Photoelectrochemistry and Photocatalytic Experiments

*Photoelectrochemical characterisation.* All the electrodes were characterised in 1 M Na_2_SO_4_ (anhydrous ≥9%, ACS reagent, Merck Group, Germany) electrolyte, prepared utilising ultrapure water (resistivity: 18.2 MΩ×cm). The electrochemical setup consisted of three electrodes connected to a Bio-Logic VSP potentiostat: Ti-NTs photoanodes were used as the working electrode (WE), Pt wire (99.997% metal basis, 0.5 mm diameter, Alfa Aesar, MA, USA) was used as the counter electrode (CE) and Ag/AgCl (SI Analytics, Mainz, Germany) was used as the reference electrode (RE). The light beam from a 500 W mercury lamp placed in an Oriel arc housing and powered with an OPS-A500 arc lamp power supply was used for irradiation (Newport, CA, USA). Two light filters, a 1.5 AM G for solar simulated light and a 420 nm cut-off filter (FSQ-GG420, Newport) for visible light irradiation, were used.

The working electrodes were made using the doctor blade method, spreading Ti-NTs paste over an area of 0.5 × 0.5 cm on a piece of FTO 2 × 1 cm and sintering the cathodes at 130 °C for 48 h. The sample paste was prepared by dispersing 100 mg of powdered sample in 200 μL of terpineol and 1 mL of acetone. The suspension was stirred and heated to 80 °C overnight [46]. A bias of 0.5 V was applied for chronoamperometry measurement. *Incident photon to the current efficiency* spectrum of (Ru(bpy)_3_)Ti-NTs was recorded by coupling the monochromator (Cornerstone 130, Newport) to the mentioned light source and applying a bias of 1 V.

*Photoreduction of methyl viologen* [47,48]. Methyl viologen bis(hexafluorophosphate) was prepared by first dissolving 514.5 mg (2 mmol) of methyl viologen dichloride hydrate 98% (MVCl_2_) (Merck) in 10 mL of ultrapure water (resistivity: 18.2 MΩ×cm) at 90 °C. At the same time, 668 mg (4.1 mmol) of ammonium hexafluorophosphate 99.98% (NH_4_PF_6_) (Merck) was dissolved in 10 mL of ultrapure water at 90 °C. The MVCl_2_ solution was added dropwise to a saturated solution of NH_4_PF_6,_ and the resulting mixture immediately became cloudy. To remove excess NH_4_^+^, the resulting solution was stirred at 80 °C for 90 min until the vapours coming from the solution were near pH 7. Finally, the solution was cooled to 4 °C and left overnight to form crystals, which were filtered and washed with ultrapure cold water. The solid was dried overnight in a vacuum at 60 °C to achieve 75% yield. To photoreduce the dicationic methyl viologen, the following procedure was applied. All samples were prepared in the same way and measured under the same conditions. A 3.05 mL solution of MV(PF_6_)_2_ with suspended catalyst was prepared in a quartz cuvette by mixing 1 mL stock solution of MV(PF_6_)_2_ (0.17 M in acetonitrile), 1 mL of titanate sample (1 mg/mL) and 50 μL of triethanolamine (TEOA) as sacrificial agent, together with 1 mL of HPLC grade acetonitrile. The dispersion was sonicated for 15 min and purged with argon for 20 min. The samples were irradiated utilising a 300 W UV-enhanced short-arc Xenon lamp, Oriel Research arc housing, and OPS-A500 arc lamp power supply. The light output was filtered through a 420 nm cut-off light filter (FSQ-GG420, Newport). The absorbance was measured by sampling 0.1 mL from the cuvette and adding it to another cuvette containing 0.9 mL of acetonitrile and 15 μL of TEOA, previously purged with argon. In all cases, the reaction baseline was corrected by measuring at zero time the absorbance of a control reaction without titanate photocatalyst. The spectrophotometer used for diffuse reflectance measurements was an Agilent Cary 300.

*Photocatalytic experiments. Hydrogen evolution reaction.* A suspension of 20 mL water-methanol solution (volumetric ratio of methanol: deionised water, 1:4) with a concentration of the solid of 0.5 mg per mL was prepared. *Photocatalytic water splitting reaction*. For all reactions, a quartz reactor was used with a volume of 50 mL. Before starting the measurements, the suspensions were sonicated for 30 min and purged with pure Ar for 30 min. Photocatalysis was performed utilising one of the three light sources. (i) Irradiation with a full UV–Vis spectrum of Hg–Xe lamp, (ii) simulated sunlight employing AM 1.5G light filter (Air Mass Filter, AM 1.5 Global, Newport), or (iii) visible light using a long pass > 435 nm cut-on optical filter (FSQ-GG435, Newport). Irradiation was performed with the 300 W Hg–Xe lamp (Hamamatsu Lightcure LC8; Prefecture of Shizouka, Japan) coupled with an optical fibre. For all cases, the irradiation energy was set in the reactor to 100 mW×cm^2^. A Hamilton syringe model 1750 SL SYR was used to sample the gas produced by the reaction and injected into a gas chromatograph previously calibrated with standards of H_2_ cylinders. *Apparent quantum yield (AQY)* measurements were carried out with a 351 ± 10 nm bandpass optical filter (FBH351-10, Thorlabs in NJ, USA), and AQY values were calculated following the literature procedure [49]. The formula utilised for this calculation can be found in the supporting information; see Equation (S1).

## 3. Results

### 3.1. Samples Characterisation

**Figure 1:**
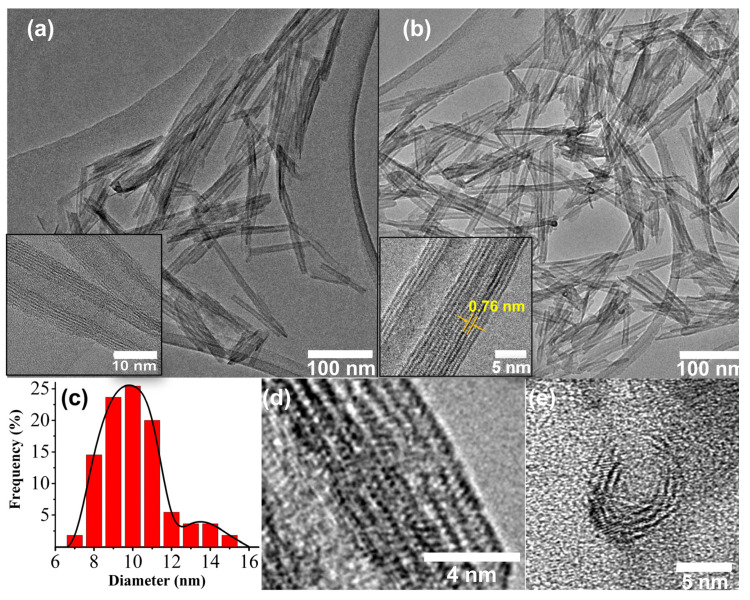
TEM micrographs of starting (Na)Ti-NTs (**a**) and produced (Ru(bpy)_3_)Ti-NTs (**b**). Top insets: HRTEM images of (Na)Ti-NTs (**a**) and (Ru(bpy)_3_)Ti-NTs (**b**) with the measured interlayer distance of (200) facet. Outer diameter distribution of (Na)Ti-NTs (**c**). HRTEM image of (Na)Ti-NT cross-section image (**d**) and along cylinder axis view of (Na)Ti-NT nanotube (**e**).

Ti-NTs samples, namely (NaH)Ti-NTs, (H)Ti-NTs, and (Na)Ti-NTs, were synthesised using a hydrothermal method as described above. TEM and X-ray powder diffraction (XRD) analysis of the three trititanates was carried out to determine the influence of the Na^+^ content on the morphology and crystal structure. TEM images of different titanates, shown in Figure 1a and Figure S1, exhibit that after hydrothermal synthesis, all anatase nanoparticles have disappeared, leaving a new composite with a high purity and nanotube morphology. These images also show that the exchange of Na^+^ cations does not affect tubular morphology. However, the XRD diffractogram shows changes in the crystalline structure of the trititanates nanotubes, see Figure S2. In the case of (NaH)Ti-NTs and (Na)Ti-NTs, XRD patterns were similar, with typical diffraction peaks of Ti-NTs appearing at 2θ values of 9.4°, 24.4°, 28.3°, 49.0° that are attributable to (020), (110), (130) and (200) facets, respectively [50]. On the other hand, XRD patterns of (H)Ti-NTs show three main changes: firstly, the suppression of the (020) facet, the decrease of intensity of the (030) peak relative to (110), and a slight shift of (200) to a smaller angle.

In order to maximise the loading of Ru(bpy)_3_^2+^ cations on the Ti-NTs, we studied the influence of Na^+^ content that affects the total amount of adsorbed Ru(bpy)_3_. For this purpose, (NaH)Ti-NTs, (H)Ti-NTs, and (Na)Ti-NTs solids were subjected to an ion exchange reaction with a concentrated solution of Ru(bpy)_3_^2+^ to form ((Ru(bpy)_3_)Ti-NTs), see Figure 1b. After ion exchange with Ru(bpy)_3_^2+,^ the nanotube morphology is maintained with no apparent changes with respect to (Na)Ti-NTs. For both samples, the measured outer diameters samples were similar to 8–15 nm (Figure 1c), and an average inner diameter ranged from 3 to 5 nm [42,51]. The average interlayer distance was measured to be around 0.8 nm for both (Na)Ti-NTs and (Ru(bpy)_3_)Ti-NTs, as shown in the insets in Figure 1a,b. The nanotube in Figure 1e is oriented perpendicular to the TEM camera and exhibits multilayer wall morphology with hollow and open-ended cylindrical structure. Unlike carbon nanotubes, titanate nanotubes are made of a single rolled titanate sheet showing typical stepped sheet packing of TiO_6_ clusters; see Figure 1d [42].

**Figure 2:**
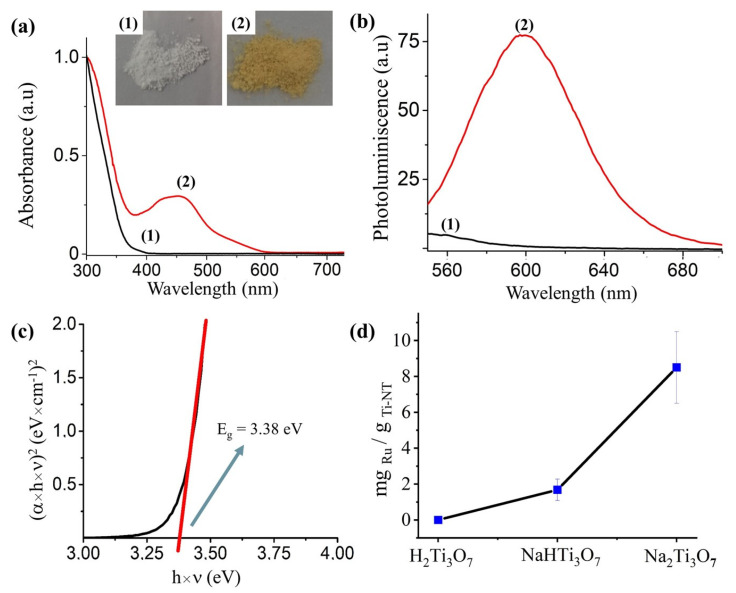
(**a**) Diffuse reflectance UV–Vis spectra and photographs of (Na)Ti-NTs (1) and (Ru(bpy)_3_)Ti-NTs (2), respectively. (**b**) Photoluminescence spectra of (Na)Ti-NTs (1) and (Ru(bpy)_3_)Ti-NTs (2). (**c**) Tauc plots for the estimation of optical band gap of (Na)Ti-NTs and (**d**) estimations of the maximum metal loading in Ti-NTs in terms of milligrams of Ru per gram of H_2_Ti_3_O_7_, NaHTi_3_O_7_ or Na_2_Ti_3_O_7_.

The ultraviolet and visible (UV-Vis) diffuse reflectance spectra of all trititanate without metallic complex were identical and resulted in white powders, showing an absorption band at 380 nm, see Figure 2a. However, the introduction of Ru(bpy)_3_^2+^ into Ti-NTs results in a yellow solid, extending absorption of the nanotubes towards the visible region, and this shift is due to the characteristic absorption band of the ruthenium bipyridyl complex centred at ~450 nm Figure 2a. Ru(bpy)_3_^2+^ has metal-to-ligand charge transfer (MLCT) transitions corresponding to ^1^(dπ^6^) → ^1^(dπ^5^π*^1^) and ^1^(dπ^6^) → ^3^(dπ^5^π*^1^); these electronic transitions take place upon excitation at the absorption band centred at 460 nm and 515 nm, respectively. After light excitation, relaxation of ^3^(dπ^5^π*^1^) excited state will occur, giving a broadband emission with a maximum of 600 nm. This broadband in Figure 2b, where the photoluminescence (Ru(bpy)_3_)Ti-NTs (2) certifies that the phosphorescence of the Ru complex is maintained by being incorporated into Ti-NTs. In addition, the quantum emission efficiency of the metallic complex in (Ru(bpy)_3_)Ti-NTs was determined to be 10 times lower than that of the molecular Ru(bpy)_3_^2+^ complex dissolved in acetonitrile. This diminution of emission efficiency suggests additional deactivation pathways of (Ru(bpy)_3_)Ti-NTs compared to the complex in solution, a fact explained by assuming electron injection from excited Ru(bpy)_3_^2+^ to the conduction band of the initial (Na)Ti-NTs. The direct optical band gap of (Na)Ti-NTs was calculated utilising the UV-Vis spectra with a value of 3.38 eV, see Figure 2c.

Elemental analysis of the different nanotubes like (NaH)Ti-NTs, (H)Ti-NTs, and (Na)Ti-NTs showed the presence of 0.4, 5.3, and 11.3 wt.% of Na, equivalent to 4, 53.41 ± 0.62 and 113.45 ± 0.54 mg of Na^+^ per gram of Ti-NT, respectively. As described in the experimental section, Ru(bpy)_3_^2+^ adsorption was carried out starting from H_2_Ti_3_O_7_, NaHTi_3_O_7,_ and Na_2_Ti_3_O_7_. Chemical analysis of Ti-NTs showed 41.74 ± 0.48 54.06 ± 0.35 mg of Na^+^ per gram of (NaH)Ti-NTs and (Na)Ti-NTs, respectively [44]. Figure 2d shows the influence of the Na^+^ content during the ion exchange process on the Ru loading (and thus Ru(bpy)_3_^2+^ loading), demonstrating that the loading depends on exchangeable Na^+^. Thus, the sample with the largest amount of Ru complex absorbed was (Na)Ti-NTs_,_ reaching a maximum Ru metallic content of 8.55 ± 0.15 mg, which means a total loading of 46 mg of Ru(bpy)_2_^3+^ per gram of sodium trititanate. In contrast, (H)Ti-NTs after ion exchange with Ru(bpy)_3_^2+^ are still colourless, and chemical analysis does not show any amount of Ru, indicating the lack of the ruthenium complex.

**Figure 3:**
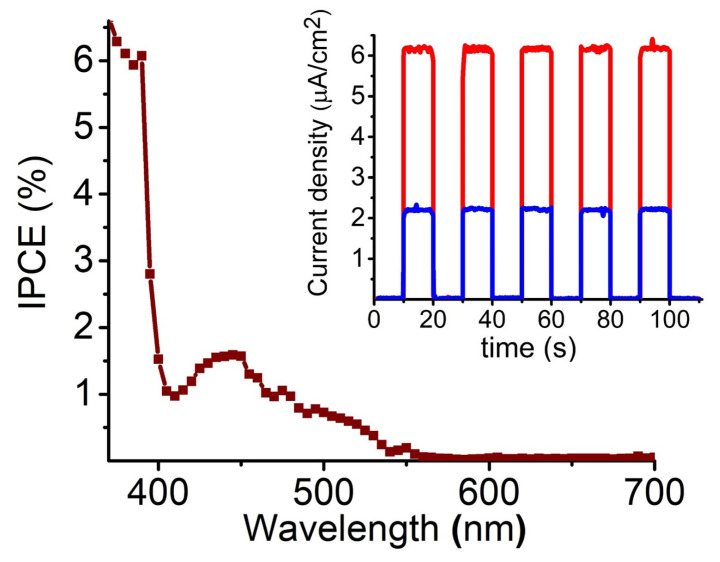
IPCE (%) vs. λ spectrum of (Ru(bpy)_3_)Ti-NTs in 1M Na_2_SO_4_ electrolyte solution irradiated with a 300 W Xe–Hg lamp and an applied potential of 1 V vs. Ag/AgCl. Inset corresponds to the photocurrent recorded with a solar-simulated light (red line) and visible light (blue line). Both photocurrent measurements were made under the same conditions using Na_2_SO_4_ as an electrolyte solution and an applied potential of 0.5 V vs. Ag/AgCl.

Electrodes made of thin films of (Ru(bpy)_3_)Ti-NTs on fluorinated tin oxide were fabricated to measure the photocurrent density response of the composite to different regions of the light spectra, as shown in Figure 3. The incident photon-to-electron conversion efficiency (IPCE) spectra of (Ru(bpy)_3_)Ti-NTs coincide very well with the optical spectrum of the solid, thus confirming current generation upon excitation of the absorption band of the adsorbed Ru(bpy)_3_^2+^ complex. These photoelectrodes of (Ru(bpy)_3_)Ti-NTs exhibited notable stability as determined by photocurrent on-off cycles, both under UV-Vis or visible irradiation, without observing significant fatigue, as seen in the inset to Figure 3.

### 3.2. Photocatalytic Activity

**Figure 4:**
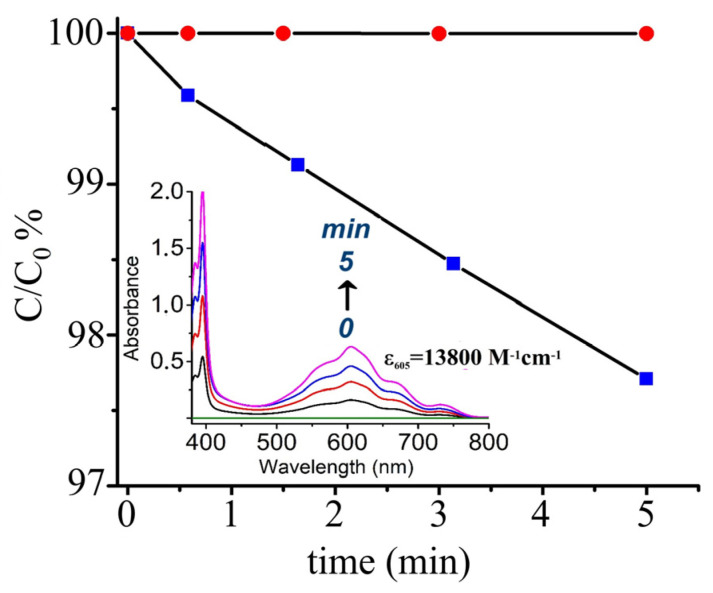
Photocatalytic activity for the reduction of MV^2+^ using (Na)Ti-NTs (red dots) and (Ru(bpy)_3_)Ti-NTs (blue squares) under visible light irradiation. The inset shows the UV–Vis spectra of reduced species of methyl viologen (MV^+·^) that grow over irradiation time.

In a preliminary experiment, (Ru(bpy)_3_)Ti-NTs were suspended in acetonitrile containing methyl viologen salt (MV(PF_6_)_2_) and triethanolamine as a sacrificial electron donor. This mixture was irradiated through a visible-light long pass (>435 nm) cut-on optical filter. The subsequent formation of MV^+^ by the reduction of MV^2+^ was measured by measuring the absorbance of the characteristic blue colour of the monovalent radical cation [47,48]. Figure 4 shows the concentration of the divalent ion as a function of time, revealing the activity of (Ru(bpy)_3_)Ti-NTs in visible light in contrast to the inactivity of the pristine (Na)Ti-NTs. The quantitative formation of MV^+^ was characterised by recording its UV–Vis absorption spectra, as seen in the inset of Figure 4. The concentration of MV^×+^ concentration was determined by measuring the optical absorbance at 605 nm and using the corresponding molar absorptivity at this wavelength of MV^+^ (ε_605_ = 13,800 M^−1^×cm^−1^, inset of Figure 4). The concentration of MV^+^ grows gradually with the irradiation time over a time scale of the order of minutes.

**Figure 5:**
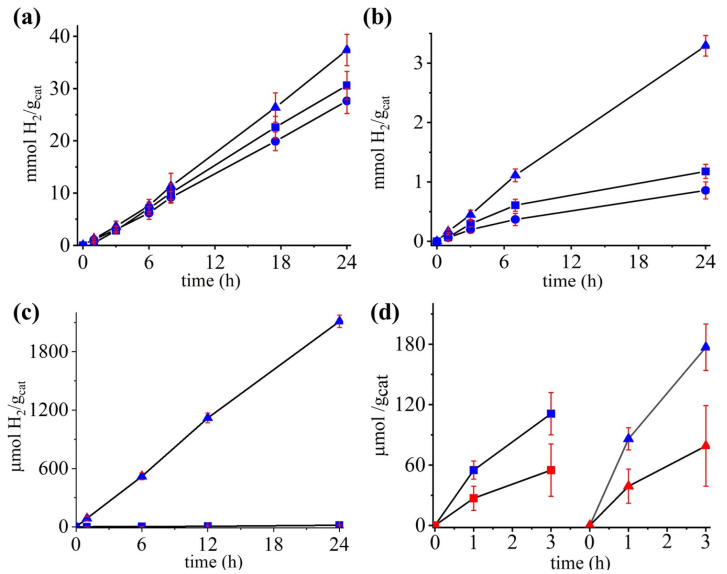
Photocatalytic H_2_ production activity upon different irradiation sources. (**a**) Irradiated with full spectra of 300 W Hg–Xe lamp and (**b**) irradiated with a solar simulator with 100mW/cm^2^ power. (**c**) Photocatalytic activity under visible light irradiation (>435 nm). (**d**) Overall water splitting in pure water, with simulated sunlight of 100 mW/ cm^2^. Codes: (▲) (Ru(bpy)_3_)Ti-NTs, (■) (Na)Ti-NTs, (●) P25. For overall water splitting, oxygen values are displayed with red symbols.

First, the photocatalytic performance of all the titanates was tested for hydrogen evolution reaction, in which an aqueous solution of 20 *v*/*v*% of MeOH as a sacrificial agent was used. The photocatalysis was studied under different irradiation sources, and TiO_2_ was used as a standard photocatalyst for this reaction (Figure 5a–c). The photocatalytic activity of (Ru(bpy)_3_)Ti-NTs, pristine (Na)Ti-NTs, and nanoparticles of TiO_2_ were compared under UV–Vis irradiation (Figure 5a) or with simulated sunlight (Figure 5b) and visible light (Figure 5c) as irradiation sources. In Figure 5a, all samples exhibit similar photocatalytic performance due to the abundance of UV light coming from the unfiltered 300 W Hg–Xe lamp. (Ru(bpy)_3_)Ti-NTs were slightly more active by reaching an H_2_ production rate of ~1.56 mmol×g^−1^×h^−1^. The photocatalytic activity of the pristine (Na)Ti-NTs is similar to P25 TiO_2_. However, under simulated solar light irradiation, the photocatalytic activity of the pristine (Na)Ti-NTs is slightly higher than that of P25 TiO_2_. It increases significantly upon the absorption of the Ru(bpy)_3_^2+^, reaching an H_2_ production rate of 3.2 mmol H_2_×g_cat_^−1^ after 24 h of reaction, see Figure 5b. In fact, the H_2_ production is about three times higher for (Ru(bpy)_3_)Ti-NTs than for the pristine (Na)Ti-NTs. To understand this difference in the photocatalytic activity, an additional experiment was carried out with the same methanol concentration in solution but using a cut-off >435 nm light filter that allows irradiation with a wavelength longer than 435 nm and the observed photocatalytic data are presented in Figure 5c. This time, it was confirmed that (Ru(bpy)_3_)Ti-NTs were able to drive the H_2_ evolution reaction, reaching an H_2_ production rate of 87.5 μmol H_2_×g_cat_^−1^×h^−1^; in contrast, under this light source, H_2_ production was negligible for (Na)Ti-NTs.

**Scheme 1:**
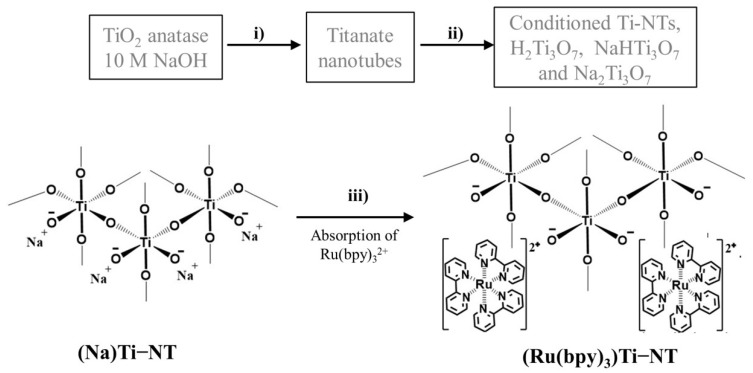
Scheme of the synthesis of [Ru(bpy)_3_]_x_Na_2−x_Ti_3_O_7_ or (Ru(bpy)_3_)Ti-NT: (i) hydrothermal treatment of TiO_2_ anatase to form titanates nanotubes. (ii) Conditioning of Na_x_H_2−x_Ti_3_O_7_ to obtain NaHTi_3_O_7_, H_2_Ti_3_O_7_, and Na_2_Ti_3_O_7_. (iii) Scheme of the preparation of [Ru(bpy)_3_]_x_Na_2−x_Ti_3_O_7_ by ion-exchange with Ru(bpy)_3_^2+^ ions.

**Figure 6:**
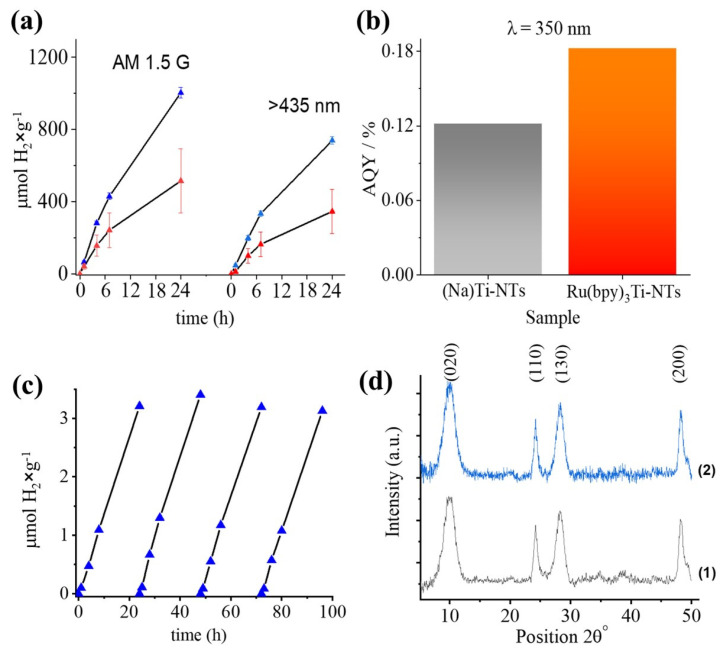
(**a**) Photocatalytic water splitting reaction of (Ru(bpy)_3_)Ti-NTs with simulated sunlight (AM 1.5G) and with visible light (>435 nm), (▲) O_2_ and (▲) H_2_. (**b**) Measurement of AQY % at 350 nm of (Na)Ti-NT in grey and (Ru(bpy)_3_)Ti-NTs in orange. (**c**) Photocatalytic stability of (Ru(bpy)_3_)Ti-NTs in the photocatalytic H_2_ evolution reaction was performed using methanol and irradiated with 100 mW/cm^2^ of simulated solar light. (**d**) XRD pattern of (1) fresh (Ru(bpy)_3_)Ti-NTs and (2) 96 h reused (Ru(bpy)_3_)Ti-NTs sample.

In addition, photocatalytic experiments were carried out to study the performance of Ti-NTs and (Ru(bpy)_3_)Ti-NTs under water-splitting conditions; in other words, in the absence of a sacrificial agent, see Figure 5d. It was found that under the same conditions, (Na)Ti-NTs produced 40% less hydrogen and oxygen than titanate photosensitised with Ru(bpy)_3_^2+^, which reached a photocatalytic production of 189 and 77 μmol×g_cat_^−1^ after 3 h of H_2_ and O_2_, respectively. This enhancement of the photocatalytic activity would be attributable to the presence of Ru dye. A long-time reaction was also tested on (Ru(bpy)_3_)Ti-NTs for 24 h to study the water splitting efficiency under solar simulated and visible light, see Figure 6a. Herein, we observed that (Ru(bpy)_3_)Ti-NTs reached a considerable H_2_ and O_2_ production after 24 h of reaction of 735 μmol×g_cat_^−1^ and 348 μmol×g_cat_^−1^, respectively, under visible light irradiation. The ability of the (Ru(bpy)_3_)Ti-NTs composite to photocatalyze the reaction under visible light is further evidence that the Ru(bpy)_3_ complex is able to promote electron transfer towards Ti-NT since it is on the trititanates where the active sites for the water-to-hydrogen reduction reaction are located (see Scheme 1). Further evidence for this fact was obtained when AQY was measured under irradiation of 350 nm, see Figure 6b. This wavelength was selected because it is a common absorption region for both titanate nanotubes and Ru(bpy)_3_. The results of AQY experiments showed that loading of Ru(bpy)_3_ afforded a benefit of 45% in the final AQY, achieving values of 0.18% for (Ru(bpy)_3_)Ti-NTs composite and 0.12% for (Na)Ti-NTs solids.

The stability of (Ru(bpy)_3_)Ti-NTs as photocatalysts was studied utilising simulated solar light by investigating four consecutive uses. Each time, the solution was refreshed using centrifugation, and the reactor was purged with argon to purge gases that could increase the accuracy of gas measurements. Similar rates of production of hydrogen were observed for consecutive experiments, as shown in Figure 6c. The XRD patterns in Figure 6d of fresh (Ru(bpy)_3_)Ti-NTs and sample used during four cycles did not show evidence of any crystallographic change. However, elemental analysis by ICP-EOS revealed the leaching of soluble ruthenium species derived from the (Ru(bpy)_3_)Ti-NTs after the four cycles experiment. After 96 h of hydrogen evolution reaction, it was found that only 3% of the total Ru content in the Ti-NTs was leached; this was considerably lower considering that after 24 h reaction in water splitting conditions, the total Ru leached was 7 ± 2%. The reason for the major leaching in the case of overall water splitting mode could be assigned to the molecular oxygen generated during the reaction that could oxidate or damage the Ru(bpy)_3_ complex. Thus, this fact could explain the slight loss of photocatalytic activity over time. Finally, the formation of RuO_2_ on the photocatalyst because of the photodegradation of the Ru complex was checked by X-ray photoelectron spectroscopy (XPS). However, no such indications were observed since the XPS spectra in Figure S3 are practically the same before and after using (Ru(bpy)_3_)Ti-NT.

## 4. Discussion

Treatment of TiO_2_ nanoparticles with concentrated NaOH solution produces trititanates nanotubes with high yield, good homogeneity, and high phase purity. According to the literature [42], crystalline TiO_2_ is decomposed into a disordered phase first, from which some (Ru(bpy)_3_)Ti-NTs type plates grow. Individual trititanate layers are peeled off from the plates and scrolled up into nanotubes. Ti-NTs are made of TiO_6_ octahedral building blocks that polymerise and form stepped sheets separated by Na^+^ or H^+^ ions located within the interlayer. The structure of Ti-NTs comprises corrugated ribbons of edge-sharing TiO_6_ octahedra. As a result of the high amounts of NaOH used during hydrothermal synthesis, the trititanates (Na_x_H_2−x_Ti_3_O_7_) have higher proportions of Na^+^ than H^+^ cations. However, these cations are easily exchangeable, making it possible to obtain Ti-NTs with different proportions of these cations in a simple process. Furthermore, using the same ionic interactions, we were able to exchange simple cations, such as Na^+^, with photoactive molecules.

TEM images and XRD patterns of the resulting Ti-NTs and (Ru(bpy)_3_)Ti-NTs showed that the nanotube morphology and crystallinity of the Ti-NTs were maintained after the ion exchange. HRTEM images of the materials showed typical hollow morphology and the multilayer configuration of Ti-NTs. This structure is in good agreement with previous studies [39,40]. Figure S1 shows the morphology of the initial Ti-NTs preserved during the ion exchange with protons or sodium cations. A similar observation occurred with (Ru(bpy)_3_)Ti-NTs, which had a lack of appreciable changes in the interlayer distance and morphology compared to initial (Na)Ti-NTs. All these facts suggest that the introduction of the metallic complex does not affect the original host structure.

Ion exchange of Na^+^ by H^+^ generates the acid form of (H)Ti-NTs and results in a variation of the XRD pattern with respect to (Na)Ti-NTs and (NaH)Ti-NTs materials (Figure S2). The loss of most Na^+^ implies a rearranged TiO_6_ unit in the crystal structure but otherwise does not affect the scroll-like morphology of the titanate. Accordingly, Teng and co-workers [52] have reported that post-treatment of (Na)Ti-NTs with acid solution generates (H)Ti-NTs specimen altering original XRD patterns. They suggested that the disappearance of the (020) peak and the increase and shift from 24.4° to 25° of the (110) peak is due to the crystalline nature of the material, which now should be composed of a mixture of anatase and protonic titanate because diffraction angles of 2θ at 25° correspond to (101) diffraction peak of anatase TiO_2_. Additionally, Zang et al. [43] have proposed that XRD pattern changes for (H)Ti-NTs are due to the dehydration of interlayered OH groups inducing the change of the crystal structure upon annealing. (Ru(bpy)_3_)Ti-NTs have the same XRD pattern as its precursor (Na)Ti-NTs, suggesting that Ru(bpy)_3_^2+^ does not affect crystalline structure upon adsorption of Ru(bpy)_3_.

In agreement with the Na^+^ content, the highest amount of ruthenium complex adsorbed on Ti-NTs was observed for (Na)Ti-NTs with 46.22 ± 0.81 mg per gram of titanate. Chemical analysis of the samples after Ru(bpy)_3_^2+^ adsorption showed a significant decrease of Na^+^ content, about 50% degree of Na^+^ exchange in (Na)Ti-NTs by the Ru(bpy)_3_^2+^complex. Apparently, even if an excess of ruthenium complex in solution was added, not all the Na^+^ cations are exchanged by the ruthenium complex, suggesting that there exists an equilibrium. Additionally, some of the Na^+^ cations are also exchanged with H^+^ naturally present in Ru(bpy)_3_^2+^ solution. The remaining amount of Na^+^ ions might not be in exchangeable positions because some Na^+^ would be responsible for preserving the nanotubular structure of Ti-NTs. The ion exchange experiments agree well with the higher ability of Na^+^ to undergo ion exchange compared to H^+^. For this reason, no Ru(bpy)_3_^2+^ absorption was observed for the (H)Ti-NTs sample. This result fits well with the known reluctance of H^+^ to undergo ion exchange due to its high charge density compared to alkaline cations.

After light excites a Ru(bpy)_3_^2+^ occur ^1^(dπ^6^) → ^1^(dπ^5^π*^1^) and (dπ^6^) → ^3^(dπ^5^π*^1^) electronic transitions, then the excited electrons must return to the ground state through several relaxation mechanisms with different deactivation kinetics that must comply Equation (1). *K_D_* is the overall excited state deactivation constant of Ru(bpy)_3_^2+^ molecules that is the result of the contributions of all the relaxation processes going on at the same time. These processes have their equilibrium constants named as of internal conversion *k_IC_*, constant for intersystem crossing *k_ISC_*, internal relaxation from singlet state *k_IR1_*, equilibrium constant of internal relaxation from first triplet excited state *k_IR2,_* and equilibrium constant of Ru(bpy)_3_^2+^ phosphorescence *k_P_*, see Scheme S1. From a phosphorescence standpoint, Figure S4 shows that the phosphorescence intensity of Ru(bpy)_3_ was 10 times lower when loaded on Ti-NTs, implying that new *k_P’_* of adsorbed Ru(bpy)_3_^2+^ is much smaller than *k_P_* of isolated molecules. The decrease of the k*_P_* value and the phosphorescence intensity of the metallic complex indicates that the electron deactivation mechanism of Ru(bpy)_3_^2+^ has been altered because of the presence of Ti-NTs. Herein, this alteration is due to the charge transfer mechanism from Ru(bpy)_3_ molecules to the Ti-NTs. Thus, adsorbed Ru(bpy)_3_ excited state deactivation kinetics must follow Equation (2), being *k_CT_* equilibrium constant of charge transfer from the metallic complex to Ti-NT. This effective charge transfer provides effective photosensitisation of Ti-NTs materials with Ru bipyridyl complexes.

(1) $$k_{IC}+k_{ISC}+k_{IR1}+k_{IR2}+k_{P}=k_{D}$$

(2) $$k_{IC^{’}}+k_{ISC^{’}}+k_{IR1^{’}}+k_{IR2^{’}}+k_{P^{’}}+k_{CT}=k_{D}$$

The current density generated during the incident photon to current efficiency experiment (IPCE, %) under light irradiation was a qualitative demonstration of the capability of the excited state of (Ru(bpy)_3_)Ti-NTs to promote a charge separation state upon light excitation. In addition to this, photocurrent response in the visible range proves that charges can flow from Ru(bpy)_3_ and thus could be used in photocatalytic reactions. For instance, photocatalytic response in the visible range in the photoreduction reaction of MV^2+^ to MV^+^ performed under visible light excitation also demonstrates that the Ru(bpy)_3_^2+^ complex is able to transfer electrons when Ru complex forms part of (Ru(bpy)_3_)Ti-NTs composite in otherwise inert Ti-NTs photocatalyst. In this experiment, the generation of MV×^+^ arises from the photoinduced electron transfer from the excited ((Ru(bpy)_3_)Ti-NTs photocatalyst to MV^2+^ as an electron acceptor, as can be seen in the supporting information as Scheme S2.

Regarding photocatalytic hydrogen evolution reaction under UV–Vis irradiation, there is only a small difference in the hydrogen production between Ru(bpy)_3_-Ti-NTs and that of the pristine (Na)Ti-NTs, being only 1.4 times higher than with standard TiO_2_ P25. This similar photoactivity is due to the high density of UV photons that are responsible for the direct photoexcitation of TiO_2_ and TiO_6_ clusters in Ti-NTs in contrast to the photoactivity observed under solar simulated or visible light, especially under simulated sunlight (Ru(bpy)_3_)Ti-NTs exceeded the photocatalytic activity in comparison to (Na)Ti-NT by almost three times, this remarkable enhancement of photocatalytic activity is derived from Ru(bpy)_3_^2+^ photosensitisation. Upon visible light photocatalysis, ((Ru(bpy)_3_)Ti-NTs generated 2100 μmol of H_2_ per gram of catalyst after 24 h, whereas (Na)Ti-NTs produced a few μmol of hydrogen. This few μmol of H_2_ detected could be attributed to the thermal decomposition of water by the presence of the sacrificial agent.

Another advantage observed in this study was the suitability of (Na)Ti-NTs photocatalysts to perform photocatalytic water splitting without a sacrificial agent [53], which could be explained due to the broader bandgap of Ti-NTs (3.38 eV) in comparison to P25 TiO_2_ (3.2 eV) [54,55]. Additionally, the position of valence and covalence bands of both materials with respect to oxidation and reduction potentials of water is relevant. From a photocatalytic point of view, this fact benefits trititanates since they have valence and covalence bands positioned towards more positive potential values, enabling the reduction of water [54]. In this regard, it was not surprising that Ti-NTs were capable of performing photocatalytic water splitting. A similar trend was also noted in the H_2_ evolution reaction and photosensitisation of Ti-NTs, leading to a 40% improvement in photocatalytic activity. Also, it was confirmed the capability of ((Ru(bpy)_3_)Ti-NTs to exploit visible light photocatalytic activity under water splitting conditions, reaching after 24 h a gas production values of 735 μmol of H_2_×g_cat_^−1^ and 348 μmol of O_2_×g_cat_. The benefit of photosensitising Ti-NTs was confirmed and measured by photocatalytic AQY % at 350 nm, leading to an improvement of 45%.

In summary, all these facts and results, plus the improvement of photocatalytic efficiency observed in the comparison of pristine (Na)Ti-NTs, confirm how photosensitisation of Ti-NTs with Ru(bpy)_3_^2+^ is a good strategy to enhance photocatalytic performance over titanates without the need of employing other sensitisers such as metallic nanoparticles or expensive co-catalyst [38,39,40]. Moreover, the present system offers the advantage of selecting among a variety of ruthenium polypyridyl complexes or other cationic dyes to further increase photocatalytic efficiency, opening the new possibility of preparing a cocktail of dyes with complementary absorption bands that could be combined to increase light absorption across almost all of the visible light spectrum.

## 5. Conclusions

This work has demonstrated that Ti-NTs, in particular with Na_2_Ti_3_O_7,_ can be photosensitised by ion exchange of Na^+^ cations with cationic dyes such as tris(2,2’−bipyridine)ruthenium(II) preserving nanotube morphology. Optical properties Ti-NTs revealed that this material has a band gap of 3.38 eV, which makes it a suitable material for photocatalytic water-splitting reactions. Photocurrent response, as well as the photocatalytic generation of MV^.+^, seems to confirm the capability of (Ru(bpy)_3_)Ti-NTs of harvesting visible light and the occurrence of photoinduced energy or electron transfer from the metallic complex to (Na)Ti-NTs. These titanates showed photocatalytic activity in both H_2_ evolution and water splitting reactions under irradiation of different light sources, including full spectra Hg–Xe lamp, simulating sunlight and visible light. Under photocatalytic H_2_ evolution reaction conditions and simulated sunlight irradiation (Ru(bpy)_3_), Ti-NTs generated 3.2 mmol×g_cat_^−1^ of H_2_ after 24 h, which is 3 and 3.5 times the photocatalytic activity of (Na)Ti-NT and TiO_2_, respectively. Also, under the same reaction conditions, photosensitised Ti-NTs exhibited 87.5 μmol H_2_×g_cat_^−1^×h^−1^, whereas pristine (Na)Ti-NTs produced no H_2_.

Ti-NTs also demonstrated the capacity to perform water-splitting reactions and again with solar-simulated light. Interestingly, (Ru(bpy)_3_)Ti-NTs had higher photocatalytic activity than (Na)Ti-NTs. The gas evolution of 187 μmol H_2_×g_cat_^−1^ and 84 μmol O_2_×g_cat_^−1^ for (Ru(bpy)_3_)Ti-NTs was observed after 3 h reaction, while the production of gases with (Na)Ti-NTs was 113 μmol H_2_×g_cat_^−1^ and 52 μmol O_2_×g_cat_^−1^. Results of the photocatalytic water splitting under visible light irradiation also confirmed the occurrence of electron transfer from the Ru metal complex to the titanate nanotubes. In fact, the benefit of loading Ru complex on Ti-NTs was confirmed with a % AQY measurement at 350 nm, resulting in an overall improvement of 45%. Finally, the stability of the material was studied in both photocatalytic reactions by performing reusability experiments. These results indicate that (Ru(bpy)_3_)Ti-NTs could be recycled four times with a minute decrease in the photocatalytic activity for the H_2_ evolution reaction without any damage to its crystallinity. However, (Ru(bpy)_3_)Ti-NTs showed leaching of 7% of total ruthenium content in water splitting reaction. In summary, our findings provide valuable insights into the effective photosensitisation of (Na)Ti-NTs, opening the field for future studies in where other metal complexes, cationic dyes, or Ru(bpy)_3_ derived complexes may be employed for photocatalytic applications.

## Data Availability

All data related to this research has been presented in the manuscript or is available in the supporting information.

## Supplementary Materials

The following supporting information can be downloaded at https://www.mdpi.com/article/10.3390/nano13222959/s1, Scheme S1: Energy scheme of electronic transitions in ((Ru(bpy)_3_)Ti-NT composite; Scheme S2: Reactions that happen during photocatalytic reduction reaction of methyl viologen; Figure S1: TEM images of three Ti-NTs with different Na^+^ and H^+^ content; Figure S2: X-Ray Diffraction of Ti-NTs with different amounts of Na^+^ and H^+^; Equation (S1): Formula employed for the calculation of the AQY %; Figure S3: X-ray photoelectron spectrum of same (Ru(bpy)_3_)Ti-NT and Figure S4. Photoluminescence spectrum of Ru(bpy)_3_ and (Ru(bpy)_3_)Ti-NT.
