# Supplementary material for: Enhanced Visible Light-Driven Photocatalytic Water-Splitting Reaction of Titanate Nanotubes Sensitised with Ru(II) Bipyridyl Complex
Source: Nanomaterials (Basel). 2023 Nov 16;13(22):2959. doi: 10.3390/nano13222959 (PMC10674862; doi:10.3390/nano13222959)
Supplement: Supplementary file 1 [file nanomaterials-13-02959-s001.zip › nanomaterials-2616531-supplementary.pdf]

Enhanced Visible−Light−Driven Photocatalytic Water−Splitting Reaction of Titanate Nanotubes sensitized with Ru(II) Bipyridyl Complex

Mauro Malizia [1], Stuart A. Scott [2], Laura Torrente-Murciano [1], Adam M. Boies [2], Talal A. Aljohani [3] and Herme G. Baldovi [1,*]

[1] Department of Chemical Engineering and Biotechnology, University of Cambridge, Philippa Fawcett Drive, Cambridge CB3 0AS, UK
[2] Department of Engineering, University of Cambridge, Trumpington Street, Cambridge CB2 1PZ, UK
[3] Refining and Petrochemical Technology Institute, King Abdulaziz City for Science and Technology, Riyadh 11442, Saudi Arabia

*Corresponding author email address: hergarba@cam.upv.es

Supporting Information

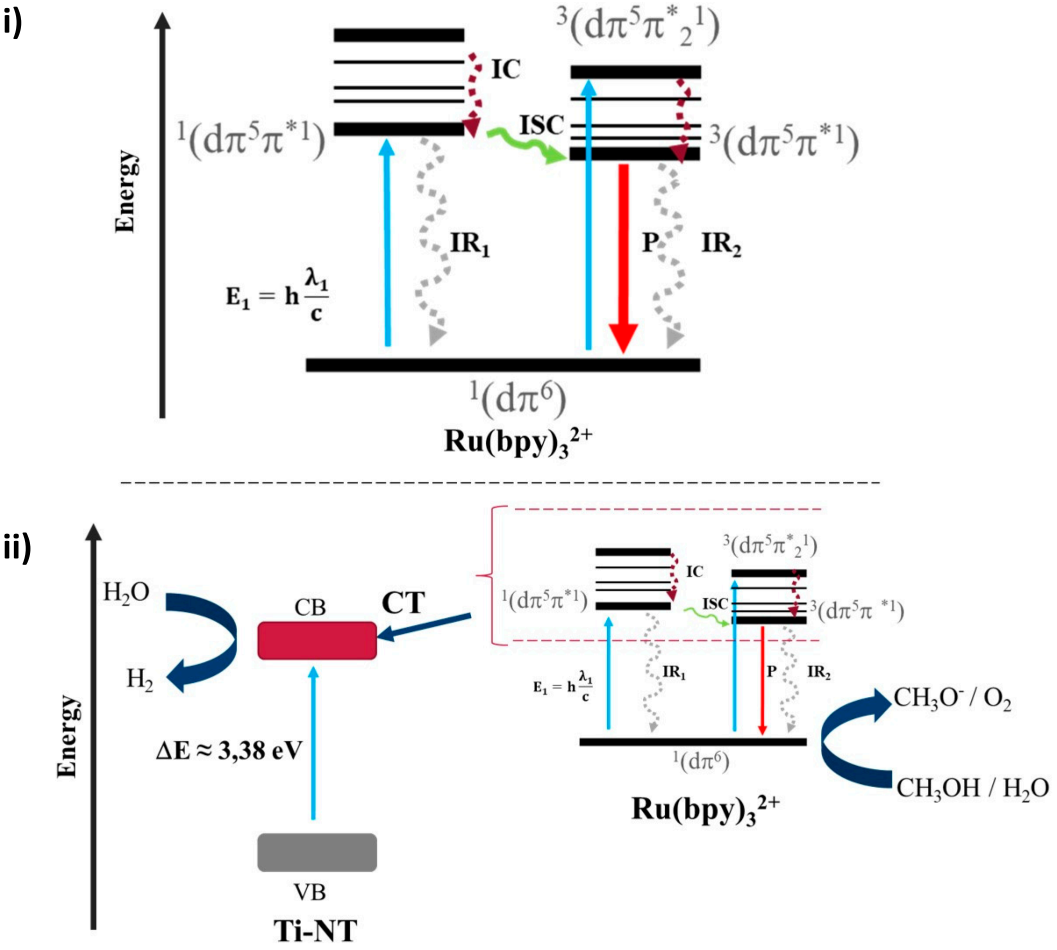

Scheme S1. h·λ/c = energy of the visible light photon. P = Phosphorescence emission. IC= Internal conversion. ISC = Intersystem crossing from $^{1}(d\pi^5\pi^{*1})$ to $^{3}(d\pi^5\pi^{*1})$. IR= Internal relaxation. CT = Charge Transfer. ***i)*** Scheme of Jablonski diagram of an isolated $Ru(bpy)_3^{2+}$. ***ii)*** Proposed energy diagram scheme of the composite $(Ru(bpy)_3)$Ti-NT when excited with visible light and performs the water splitting reaction.[1]

$$\mathrm{Ru(bpy)_3{*}\text{-}(Ti\text{-}NT)} \xrightarrow{>420\ nm} \mathrm{Ru(bpy)_3{*}\text{-}(Ti\text{-}NT)} \longrightarrow \mathrm{Ru(bpy)_3\text{-}\ (Ti\text{-}NT)\ {*}} \quad (1)$$

$$\mathrm{Ru(bpy)_3\text{-}\ (Ti\text{-}NT)\ {*} + MV^{2+}} \longrightarrow \mathrm{MV^{+} + Ru(bpy)_3\text{-}\ (Ti\text{-}NT)^{+}} \quad (2)$$

$$\mathrm{Ru(bpy)_3\text{-}\ (Ti\text{-}NT)^{+} + N(C_2H_5OH)_3} \longrightarrow \mathrm{N(C_2H_5OH)_2\ C_2H_5O^{-} + Ru(bpy)_3\text{-}\ (Ti\text{-}NT)} \quad (3)$$

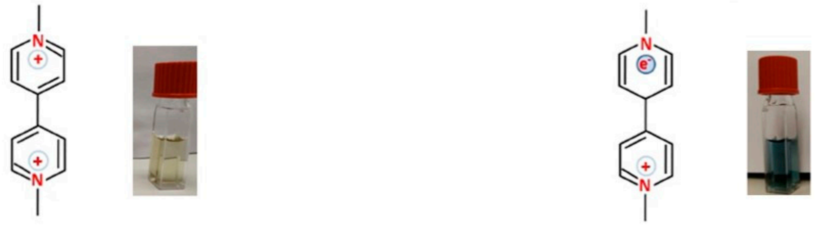

Scheme S2. Reactions that happens during photocatalytic reduction reaction of methyl viologen by ($Ru(bpy)_3$)Ti-NTs utilizing visible light. Equation (1) is the photoexcitation of ruthenium complex with visible light followed by charge transfer to Ti-NT, equation (2) is the methyl viologen reduction reaction with excited form of ($Ru(bpy)_3$)Ti-NTs and equation (3) corresponds to the reaction of the oxidant pair with the sacrificial agent tritethanolamine, (TEOA). Images on the bottom: on the left, image corresponds to the draw of dication methyl viologen (MV^{2+}) and a photograph of a fresh solution of MV^{2+} that is yellowish-transparent and on the right, the draw and cuvette the blue liquid corresponding to the reduced specie of methyl viologen ($MV^{\cdot+}$).[2]

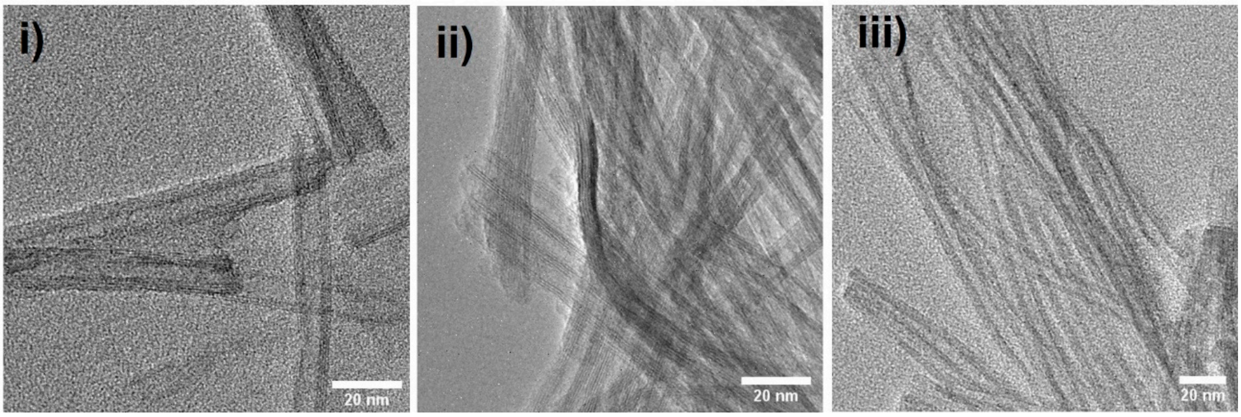

Figure S1. TEM images of titanates nanotubes conditioned at different (i) (H)Ti-NTs, (ii) (NaH)Ti-NTs and (iii) (Na)Ti-NTs.

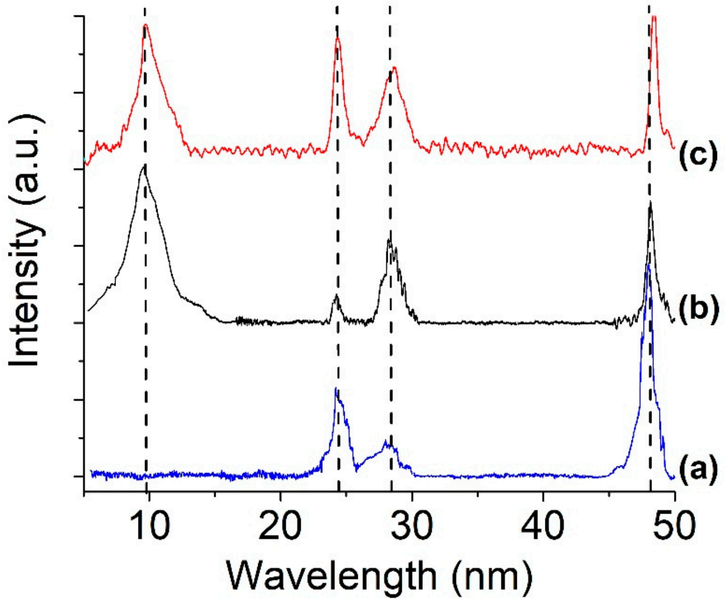

Figure S2. Experimental X-Ray diffraction patterns of the trititanate nanotubes samples: (a) (H)Ti-NTs, (b) (NaH)Ti-NTs and (c) (Na)Ti-NTs. The pics at 9.6°, 24.4°, 28.3°, and 48.° are relate to the titanate nanotubes facets (020), (110), (130) and (200) planes respectively.[3], [4]

$$\mathbf{AQY\ \%} = \frac{\boldsymbol{e \cdot n \cdot N_A \cdot c \cdot h}}{\boldsymbol{P \cdot t \cdot S \cdot \lambda_{inc}}} \cdot \mathbf{100}$$

Equation S1. Formula employed for the calculation of the AQY % that derived from AQY % = E x R/I that means E electrons, R reaction rate and I incident photon flux (e) number of electros needed to produce the molecule, (n) amount of formed H_2 in mol/time, (N_A) Avogadro number in molec/mol, (c) speed of light in m/s, (h) as Planck's constant in J·s, P power density of the incident light in J/s·cm^2, (S) is the incident light surface in the reactor in cm^2, (t) corresponds to the time of reaction in s and λ_{inc} as incident light wavelength .

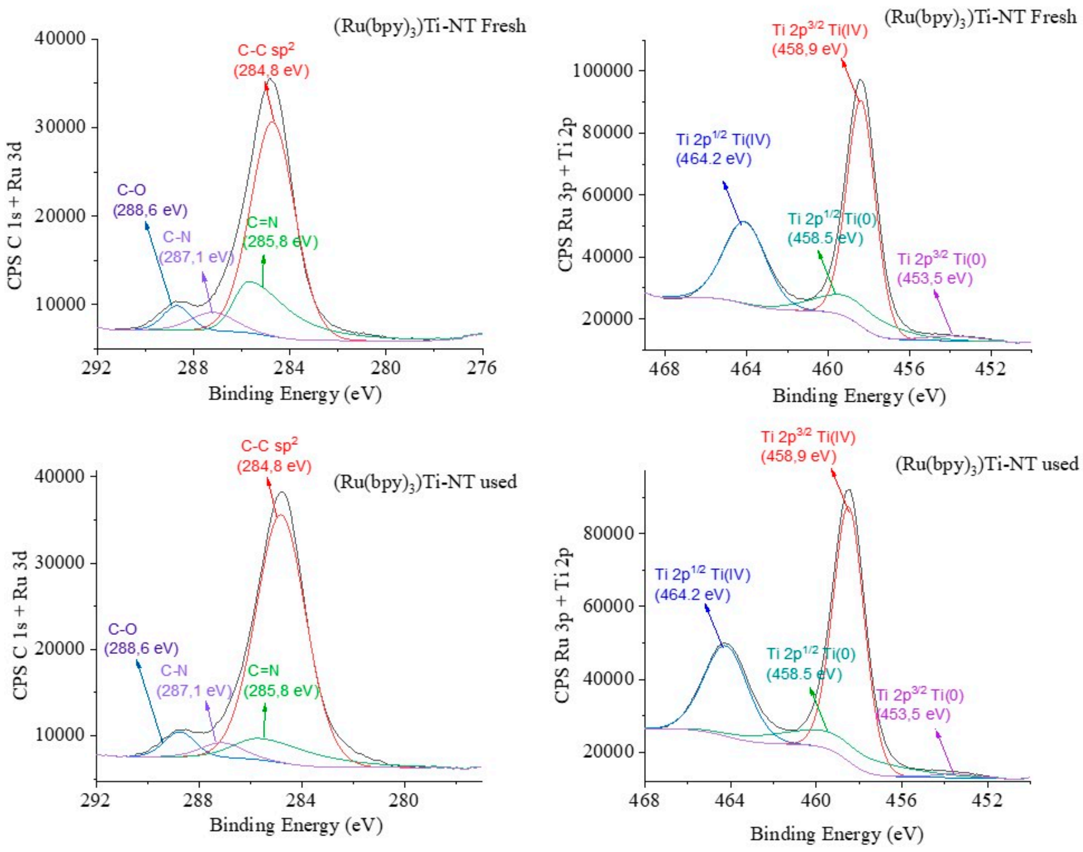

Figure S3. X-ray photoelectron spectrum of same ($Ru(bpy)_3$)Ti-NT sample before (fresh) and after (used) overall water splitting photocatalysis

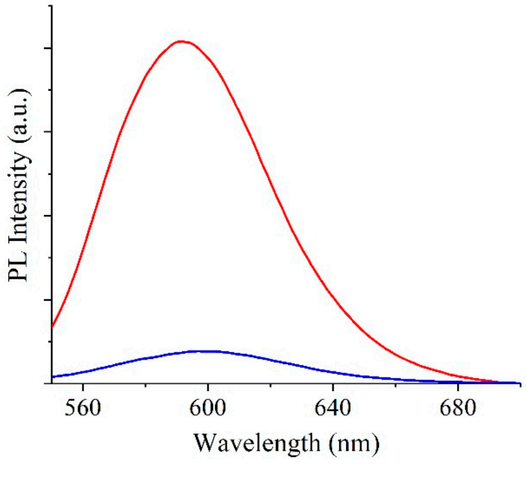

Figure S4. Photoluminiscence spectrum of the phosphorescence of $Ru(bpy)_3$ in red and ($Ru(bpy)_3$)Ti-NT in blue after excitation at 450 nm. Both samples had same absorbance at 450 nm.

References.

[1] "[Ru(bpy)3]2+* and other remarkable metal-to-ligand charge transfer (MLCT) excited states ," *Pure and Applied Chemistry* , vol. 85. p. 1257, 2013.

[2] T. W. Ebbesen, G. Levey, and L. K. Patterson, "Photoreduction of methyl viologen in aqueous neutral solution without additives," *Nature*, vol. 298, no. 5874, pp. 545–548, 1982.

[3] Q. Chen, W. Zhou, G. H. Du, and L.-M. Peng, "Trititanate Nanotubes Made via a Single Alkali Treatment," *Adv. Mater.*, vol. 14, no. 17, pp. 1208–1211, Sep. 2002.

[4] M. Zhang *et al.*, "Effect of annealing temperature on morphology, structure and photocatalytic behavior of nanotubed H2Ti2O4(OH)2," *J. Mol. Catal. A Chem.*, vol. 217, no. 1, pp. 203–210, 2004.
